# Advancing 3D printed microfluidics with computational methods for sweat analysis

**DOI:** 10.1007/s00604-024-06231-5

**Published:** 2024-02-27

**Authors:** Emre Ece, Kadriye Ölmez, Nedim Hacıosmanoğlu, Maryam Atabay, Fatih Inci

**Affiliations:** 1 UNAM-National Nanotechnology Research Center, Bilkent University, 06800 Ankara, Turkey; 2 Institute of Materials Science and Nanotechnology, Bilkent University, 06800 Ankara, Turkey; 3 Department of Chemistry, Hacettepe University, 06800 Ankara, Turkey

**Keywords:** 3D printing, Microfluidic chips, Sweat analysis, Density functional theory, Biosensor

## Abstract

The intricate tapestry of biomarkers, including proteins, lipids, carbohydrates, vesicles, and nucleic acids within sweat, exhibits a profound correlation with the ones in the bloodstream. The facile extraction of samples from sweat glands has recently positioned sweat sampling at the forefront of non-invasive health monitoring and diagnostics. While extant platforms for sweat analysis exist, the imperative for portability, cost-effectiveness, ease of manufacture, and expeditious turnaround underscores the necessity for parameters that transcend conventional considerations. In this regard, 3D printed microfluidic devices emerge as promising systems, offering a harmonious fusion of attributes such as multifunctional integration, flexibility, biocompatibility, a controlled closed environment, and a minimal requisite analyte volume—features that leverage their prominence in the realm of sweat analysis. However, formidable challenges, including high throughput demands, chemical interactions intrinsic to the printing materials, size constraints, and durability concerns, beset the landscape of 3D printed microfluidic devices. Within this paradigm, we expound upon the foundational aspects of 3D printed microfluidic devices and proffer a distinctive perspective by delving into the computational study of printing materials utilizing density functional theory (DFT) and molecular dynamics (MD) methodologies. This multifaceted approach serves manifold purposes: (i) understanding the complexity of microfluidic systems, (ii) facilitating comprehensive analyses, (iii) saving both cost and time, (iv) improving design optimization, and (v) augmenting resolution. In a nutshell, the allure of 3D printing lies in its capacity for affordable and expeditious production, offering seamless integration of diverse components into microfluidic devices—a testament to their inherent utility in the domain of sweat analysis. The synergistic fusion of computational assessment methodologies with materials science not only optimizes analysis and production processes, but also expedites their widespread accessibility, ensuring continuous biomarker monitoring from sweat for end-users.

**Graphical Abstract:**

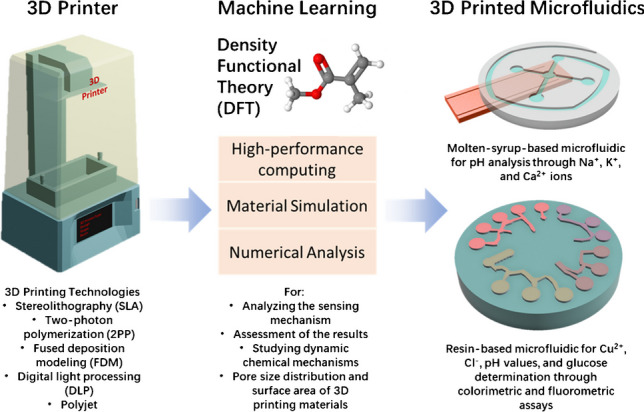

## Introduction

**Fig. 1:**
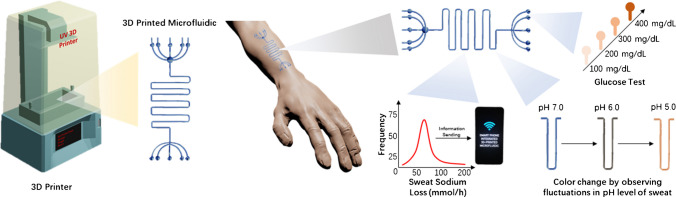
Schematic illustration of the 3D printed microfluidic platform and the application of such platforms to skin for measuring multiple parameters and biomarkers from sweat

In recent years, humanity has fervently embraced technologies that empower individuals to scrutinize their health status [1]. Particularly, glucose sensors stand as vanguards in this wave of transformative technological progress [2]. Traditionally, the analysis of target molecules was confined to laboratory settings before becoming accessible to the end-users. However, with the evolution of biosensor technologies, these analyses can now be conveniently conducted in domestic environments, thereby effecting significant savings in both cost and time [3, 4]. Analysis of sweat, a complex biological fluid containing various analytes, is crucial for tracking health conditions [5, 6] such as drug and alcohol monitoring [7], temperature regulation [8], infectious diseases [9], glucose levels [10], and hydration levels [11] (Fig. 1).

During the metamorphosis of bulky biosensing systems into their portable versions, microfluidic systems have taken a crucial role since they offer a broader range of miniaturized total chemical analysis systems based on the manipulation of fluidic devices in micro/nanoscale volumes [12, 13]. Investigations of microfluidic devices and their fabrication with micromechanics technology back in the 1970s first launched with gas chromatograph and inkjet printer nozzles, then continued with flow sensors and valves to complex microfluidic systems for chemical and biological analysis. Various fabrication methods have been employed for microfluidic device fabrication and continually developed [14, 15]. The availability of material, environmental impact, device dimension, cost, and rapid fabrication process are critical parameters affecting overall device performance and wide application [16]. Among them, 3D printing technologies are emerging advancements with varied techniques, including stereolithography (SLA) [17], two-photon polymerization (2PP) [18], fused deposition modeling (FDM) [19], digital light processing (DLP) [20], and PolyJet [21] techniques which are adaptable methods for microfluidic device fabrication. The materials selection depends on which 3D printing techniques are employed, including plastic (acrylonitrile, 1,3-butadiene, and styrene (ABS) [22], poly methyl methacrylate (PMMA) [23], and poly lactic acid (PLA) [24]), metal (titanium [25] and Inconel 625 [26]), and composite materials (carbon fiber [27] and fiberglass [28]). Even though many of these materials cannot be used directly, they are added to the printing material to increase the materials’ heat capacity or mechanical strength [29, 30].

In addition, one of the strongest points of these 3D printed microfluidic devices, which can be produced quickly and at a low-cost, is that they can be customized to the person’s requirements. Moreover, the properties of printing materials, including flexibility [31], optical transmittance [32], and heat and mechanical resistance [29, 30], can be altered and produced by including additives or adjusting device settings (such as exposure time and resolution) in the direction of interest. In addition to improving the quality of these materials, properties such as pore width, flexibility, and mechanical strength of materials are made possible by analyzing them at the molecular level, owing to computational techniques such as DFT [33, 34]. Moreover, the sensor system (tattoo [35] and pH monitoring [36]) to be developed depending on the target molecule can be included in these microfluidic systems at the end of the day. By adding electronic circuits to these systems, information flow can be instantly transferred to devices such as smartphones via NFC, Wi-Fi, or Bluetooth [37, 38].

Focusing on the adaptation of health monitoring systems for daily use, low-cost, short turnaround time, and ease of use are pivotal parameters for the end-users. Therefore, 3D printed microfluidic devices for sweat analysis would be a niche for this manner. Moreover, blood drawing requires invasive methods such as needles. Since sweat is on the body’s surface, it does not require using any needles or other invasive methods [39], and this is an appealing point for patients, newborns, children, and those who are having difficulties in blood sampling [40]. Briefly, sweat is a rich source of biomarkers including ions (sodium (Na^+^), potassium (K^+^), and chloride (Cl^−^)), metabolites (lactate, glucose, and urea), hormones (cortisol and testosterone), metals (arsenic (As) and lead (Pb)), and enzymes (amylase and creatine kinase) in which are secreted from the sweat glands in the dermis and secreted through the dermal duct from dermis to the uppermost layer of the epidermis to the skin [41]. Changing conditions of the body fluids are also reflected to the sweat since the sweat secretion is an ultimate route of channeling toxins and disposed molecules out of the body. This rich mixture of components makes sweat as an ultimate source of biomarker detection related to different diseases [42, 43]. Moreover, continuous monitoring can be implemented by constantly monitoring sweating. To exemplify, Nyein et al. managed to continuously and autonomously monitor the body’s pH, Cl^−^, and levodopa levels by developing a wearable patch. By this means, it was possible to watch stress situations, activities, and findings associated with Parkinson’s disease instantly [44]. Many different studies have revealed that the amounts of glucose and lactate in blood are strongly related to the levels of molecules in sweat [40]. To simply put the combination of the ability to perform multiple analyses using sweat and the advantages of those mentioned above, 3D printed microfluidic devices help to put forward a promising health monitoring system.

This review aspires to undertake a thorough exploration of the application of 3D printed microfluidic systems integrated with biosensors for detecting biomarkers in sweat. Moreover, our endeavor extends to bridging the divide between these platforms and computational perspectives by elucidating select computational studies focused on the materials integral to 3D printing for advancing microfluidic systems for health monitoring. In this regard, computational strategies would enhance comprehension of the intricacies inherent in microfluidic systems, facilitate in-depth and comprehensive analyses, concurrently economize both cost and time, refine the optimization of design elements, and bolster the resolution. The studies mentioned below are directed towards propelling significant strides in enhancing the manufacturability and adaptability of 3D-printed microfluidic systems, with the ultimate aim of meeting the discerning needs and expectations of the end-users.

## 3D printing devices for fabricating microfluidic systems

Conventional microfluidic fabrication methods heavily depend on expensive and sophisticated cleanroom facilities, including soft photolithography steps that are time-consuming and tedious processes [45]. On the other hand, 3D printing, as a revolutionary additive manufacturing technology, provides a simplified, facile, and accessible method for fabricating microfluidic devices in one or two steps [46]. Contrary to the traditional methods, 3D printing minimizes the labor of the fabrication process, eliminating cleanroom setups and reducing the time required to fabricate microfluidic systems. 3D printing in microfluidics allows complex, robust, and intricate structures. 3D printing methods (SLA, 2PP, FDM, DLP, and PolyJet) offer the rapid production [47] of microfluidic devices, forming complex channel structures, precise control over dimensions, and several materials, including tailored resins for diverse microfluidic applications [48]. Various microfluidic devices with different functionalities can be fabricated, including 3D microfluidics for cell culture [49], biosensing [50], drug delivery, inertial microfluidics [51], micromixers [16], and droplet-based microfluidics [52].

**Fig. 2:**
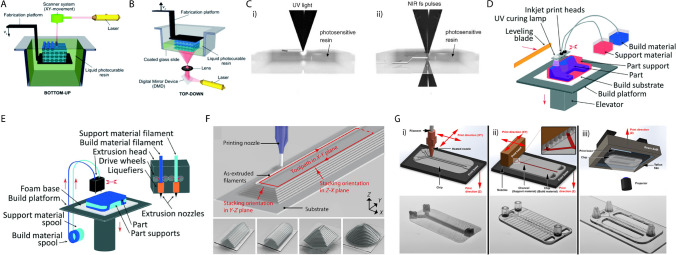
Schematic illustrations of 3D printing techniques. (**A**) free and (**B**) constrained configurations of SLA 3D printing systems. Reprinted with permission [55]. Copyright 2022, Royal Society of Chemistry. (**C**) one-photon laser (i) and two-photon laser (ii) absorption.  Reprinted with permission [67]. Copyright 2006, Elsevier. (**D**) PolyJet 3D printing system [54]. (**E**) FDM 3D printing system. Reprinted with permission [54]. Copyright 2016, Royal Society of Chemistry. (**F**) Layer-by-layer printing. Reprinted with permission [58]. Copyright 2020, Science Advances. (**G**) Comparison of roughness and resolution in microfluidics fabricated by SLA-DLP (i), PolyJet (ii), and FDM (iii) 3D printing. Reprinted with permission [56]. Copyright 2017, American Chemical Society (ACS)

Briefly, SLA employs a focused UV laser to selectively cure layers of liquid photopolymer resin to build the desired structures by controlling the positions of the laser focus, layer-by-layer, repeatedly and gradually. SLA provides delicate, intricate structures with high resolution and smooth surfaces, but it is limited to a slow speed due to single-point laser operation [53]. SLA can be operated either in free (bath) or constrained (bat) configurations, as demonstrated in Fig. 2A, B. In the free surface method, a mobile build platform is submerged in a tank with photo-curable resin, while the constrained surface method utilizes a platform above the resin tank cured with UV light. DLP works similarly to SLA, yet a light source is utilized instead of a UV source to cure photopolymer resin. It is faster than SLA due to the simultaneous curing of all layers at once. In the 2PP technique, a femtosecond laser is employed to cure photopolymers similar to SLA. However, it can fabricate a volume structure without a layers-by-layer technique due to a photoinitiator’s absorption of two photons. The representation of SLA that applies a one-photon laser and two-photon laser of 2PP is depicted in Fig. 2C. Although high resolution is achieved and many materials could be adaptable, it is a slower technique than SLA and FDM.

As a second method, PolyJet printing operates by jetting a liquid photosensitive polymer ink to deposit layers and curing with UV light to obtain desired structures. This method has the potential for mass production and enables complex multi-material fabrication, including rigid and flexible forms. Employing diverse materials enables the creation of objects with different properties, like soft and hard plastics, elastomers, and different colors. Its high resolution and multi-material properties make more attractive for the applications of microfluidic devices. However, the tedious post-process of cleaning the sacrificial layer and the cost are the main limitations to boosting its applicability [54].

FDM technology is commonly used for 3D printing; a thermoplastic is melted and extruded through a heated nozzle and deposited layer by layer to build a microfluidic structure [55]. Despite the fact that it is widely used with many thermoplastic polymers, the resolution and strength of structures are low due to incomplete layer fusion. FDM is cost-effective and straightforward; however, it is limited to low precision and pore formation compared to SLA and DLP techniques.

The working principles of SLA, PolyJet, FDM, and layer-by-layer 3D printing technologies, along with their respective components, are depicted in Fig. 2. A comprehensive study is illustrated in Fig. 2G, which evaluates microfluidic performance printed by SLA-DLP, FDM, and PolyJet 3D printing techniques. It has been shown that SLA-DLP offers less roughness, making it more suitable for precise flow control. On the other hand, SLA-DLP and PolyJet provide high resolution according to FDM techniques [56]. Although 3D microfluidic-based printing is a promising fabrication method, it has yet to reach the level of resolution required by lithographic techniques. 3D printing technology for microfluidic devices with high precision is in the developmental stage, and further advancements in materials science, optimization processes, and printing techniques are necessary to expand its applicability.

## Materials for 3D printed chips

Various thermoplastic polymers, photopolymers, and elastomers can be implemented for 3D printing microfluidic devices [57, 58]. The choice of materials holds significant importance within microfluidics, influencing critical aspects such as resolution, mechanical strength, transparency, biocompatibility, surface quality, and manipulation of fluid flow. Each of these parameters plays a pivotal role that impacts the overall functionality and performance of the microfluidic application. As an example, polydimethylsiloxane (PDMS) is a widely used elastomer in microfluidics due to its inertness, robust structure, and transparency, which is conventionally utilized with soft lithography for microfluidic fabrication [55]. 3D printing enables the fabrication of PDMS either directly or as a mold [59]. The 3D printing for PDMS as a mold is reported down to a 10-µm feature resolution [60]. Fabrication of microfluidic devices employs a series of lithography techniques. A photomask is generated initially by soft lithography to transfer the pattern to SU8 spin-coated silicon wafers. Following the pouring and peeling of PDMS, the glass substrate is encapsulated with PDMS via oxygen plasma bonding. In contrast, 3D presents a simple approach and eliminates multistep fabricating a PDMS as a mold or direct fabrication of a complete microfluidic device while retaining its oxygen permeability and biocompatibility [53]. The PDMS mold and the whole microfluidic device can be fabricated using SLA, DLP, FDA, and PolyJet 3D printing techniques.

Thermoplastic is a sub-division of polymers that soften when heated and solidify when cooled. Thermoplastics are reprocessable and do not undergo irreversible changes, unlike thermosetting polymers, which allow them to be melted, reshaped, and solidified many times while retaining their properties. Almost all thermoplastic can be printed by FDM technology. There are numerous commercial thermoplastics that can be employed in 3D printing, including PMMA, PLA [61], polycarbonate (PC) [62], ABS [63], polypropylene (PP), and polyethylene terephthalate glycol (PETg). Among them, ABS and PLA polymers are widely utilized for robust microfluidic systems due to their durability and mechanical strength. Besides direct printing of microfluidic devices, PLA and ABS-based molds can be printed via FDM printing [64].

Photopolymerization is a chemical reaction triggered by photosensitive material when exposed to light, generally a UV or visible light wavelength. Hence, it undergoes a process where the molecules within polymer material react and form large molecule structures. Acrylates, epoxides, and urethanes are mainly photopolymers that have been used, and they are prone to swelling in some solvents, which inhibits their wide range of applications [65]. In addition, SLA, DLP, and 2PP, PolyJet technologies, utilize photopolymers [66].

## Sweat analysis through biosensors integrated with 3D printed chips

**Fig. 3:**
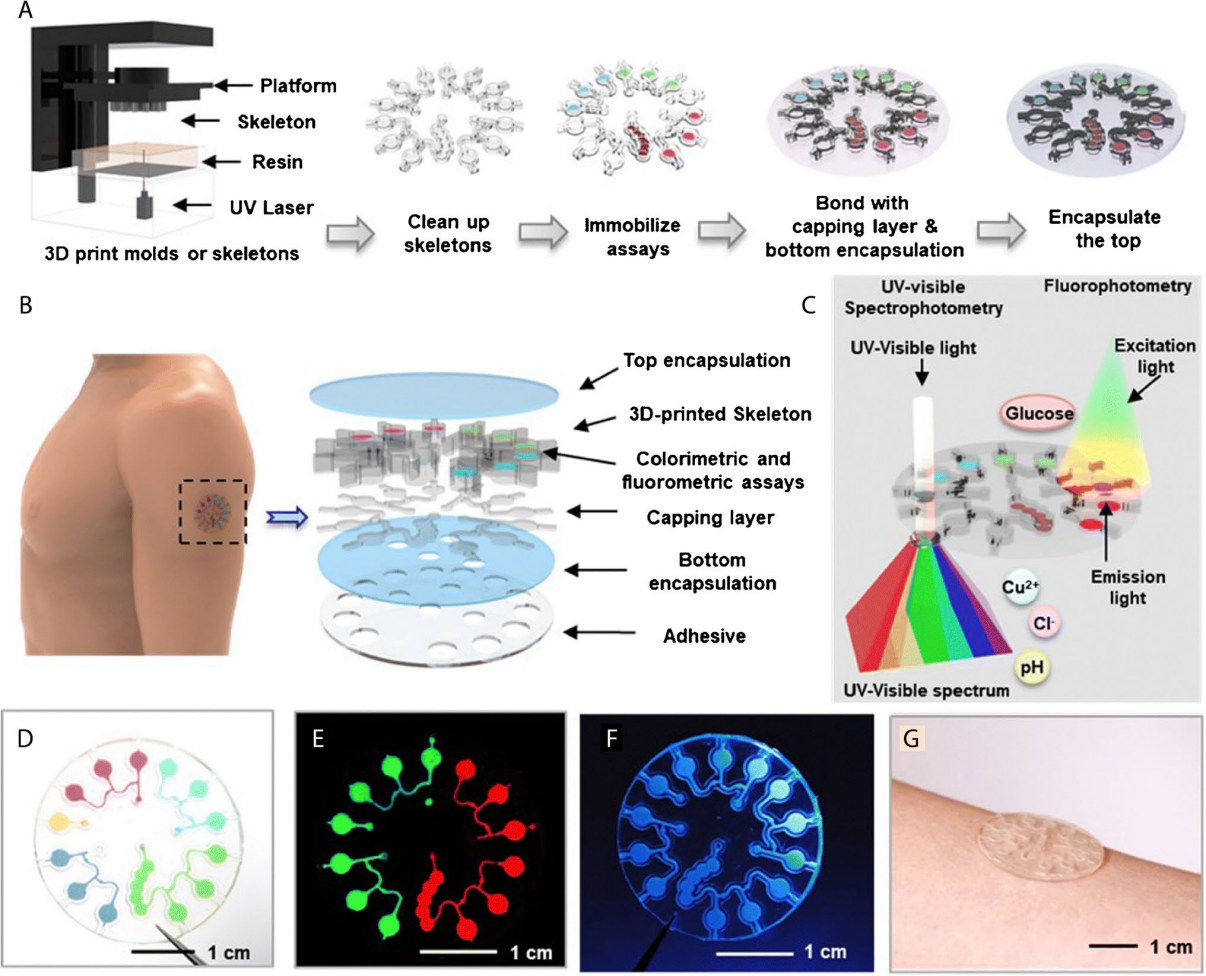
(**A**) Fabrication steps of 3D printed microfluidic sweat analysis device is depicted. (**B**) The layers of the device, (**C**) the analysis methods for the target analytes, (**D**−**F**) internal structure of the device with different dyes, and (**G**) the completed device are presented. Reprinted with permission [70]. Copyright 2023, Royal Society of Chemistry

The ongoing literature effort has turned into a few designs that could integrate 3D printed microfluidic platforms as sweat analysis platforms combined with in-device biosensors. In a pioneering study, researchers demonstrated one of the first examples of 3D printed microfluidic chips [68]. The molten-syrup-based 3D printing technique used in this study is an eco-friendly and low-cost alternative to plastic-based 3D printing materials. Positive molds created with molten-syrup printing were later complemented with PDMS and additive layers with embedded electrodes to form the final skin mounting device for sweat collection and analysis. Integrated electrochemical sensor to the system with electrodes resulted in the comparative readout of sweat pH values compared with a commercial pH-meter in a narrow measurement range and real-time fashion. In another study, a 3D printed microfluidic sampling module with a flexible and multiplex sensor module patch from an integrated circuit is demonstrated [69]. Overall, the design in this study is capable of monitoring multiple electrolytes (Na^+^, K^+^, and Ca^2+^) from non-invasively collected sweat samples. As a result, all three target ions could be detected between 0.1 and 100 mM concentration using the silver 3D printed electrode including ion-selective membrane. In order to analyze more indicators from sweat, Yang et al. demonstrated a multiplexed sweat analysis module based on spectroscopic measurement [70]. This study initially investigates the properties of flexible 3D printing resins by printing a microfluidic design with sweat containers and reaction chambers in order to achieve an ideal measurement chamber for the optic components. Next, the reaction chambers were filled with either colorimetric assays for the concentration determination of Cu^2+^, Cl^−^, and pH values or a fluorometric reaction for glucose concentration detection (Fig. 3A). The resulting system, which is attached with a medical-grade adhesive to the skin, shows compatible results in human trials with laboratory results on a scale of 0–3 ppm for copper, 0–160 mM for Cl, pH 4.0 to pH 9.0, and 0–50 µM for glucose.

Additionally, 3D printing could be used not only to design microfluidic systems, but also measurement chambers for sweat collection studies. In a different study, a smartphone-based lactate measurement system was developed based on lactate oxidize reaction and detecting hydrogen peroxide output with horseradish peroxidase (HRP) luminometric assay. The overall system was composed of a mini cartridge that contains the reactions, a dark box to contain light output generated by HRP-luminol reaction, and a universal holder for smartphones [71]. The designed device is capable of measuring lactate levels in sweat and saliva with the millimolar level per liter in both types of samples. Apart from being a production platform for microfluidic chips, 3D printing could also assist microfluidic system production for sweat analysis by being a low-cost and high-volume platform for mold production. In Nah et al. (2021) study, scientists utilized this advantage of 3D printing by designing a flexible microfluidic system based on PDMS reaction chambers and Ti3C2Tx MXene-loaded laser-burned graphene as flexible electrode material on flexible polyimide (PI) film, for later also to be transferred to PDMS [72].

Considering the advancement of the 3D printing field and the advantages of the overall approach as being a low-cost, easy-to-produce, and easy-to-deploy alternative to the current methods, 3D printed microfluidic systems for sweat collection and on-chip analysis will be a game changer in the future. Moreover, the role of computational studies can support game changer platforms by analyzing and predicting 3D printing materials.

## Computational methods in designing materials for 3D printing and advancing performance of sensors

In the past decade, novel technologies have been developed and applied by researchers for biomarker detection. Reactions extensively happen between analytes in solution and receptors on a surface [73]. Microfluidic systems include continuous flow regimes in micron-sized channels that are developed for different biological and chemical applications [74]. Due to the limitation of experimental methods at the microscopic level, a combination of in silico designs with experimental studies has immense potential in developing biosensors [75]. This part of the review exhibits an overview of the applications of computation methods, particularly DFT to study and the design of 3D printed microfluidic biosensors. Furthermore, for more clear understanding of these methods, Table 1 briefly presents some comparisons and properties of computational and simulation methods.

**Table 1:** Comparisons and properties of computational methods

Density functional theory (DFT)	Theory	Solving of Kohn–Sham equations for many-body systems [76, 77]
	Applications	• Electronic and magnetic properties of semiconductors • Prediction of mechanical properties • Prediction of sensitivity of nanostructures to environmental pollutants • Prediction of molecular properties, geometries, total energies, and vibrational frequencies [76, 78–80]
	Software	GAUSSIAN, MOPAC, ABINIT, Quantum Espresso, OpenMx, Qbox, VASP, ORCA, GAMESS, and SIESTA [81]
Molecular dynamics (MD) simulation	Theory	Solving of Newton’s equation of motion for atoms and molecules [82]
	Applications	• 3D structures of macromolecules • Conformation of ligand–protein interaction • Drug design and drug delivery • Characterization of membrane structure and organization • Lipid–drug interactions • Lipid–protein interactions • Protein structure and dynamics • Membrane permeability [83, 84]
	Software	DL_ POLY, AMBER, NAMD, LAMMPS, GROMACS, CHARMM, and TINKER [85]
Molecular docking	Theory	Using a search algorithm in prediction of interaction energies of ligand and receptor [86–89]
	Applications	• Identifying ligand binding pocket • Prediction of ligand–protein interactions • Prediction of protein–protein interactions • Prediction of pollutants degraded by enzymes • Drug design [90, 91]
	Software	Swissdock, Autodoc Vina, Dock, ZDOCK, AutoDock, Glide, Hdock, Gold, and Flex X [92–94]

**Fig. 4:**
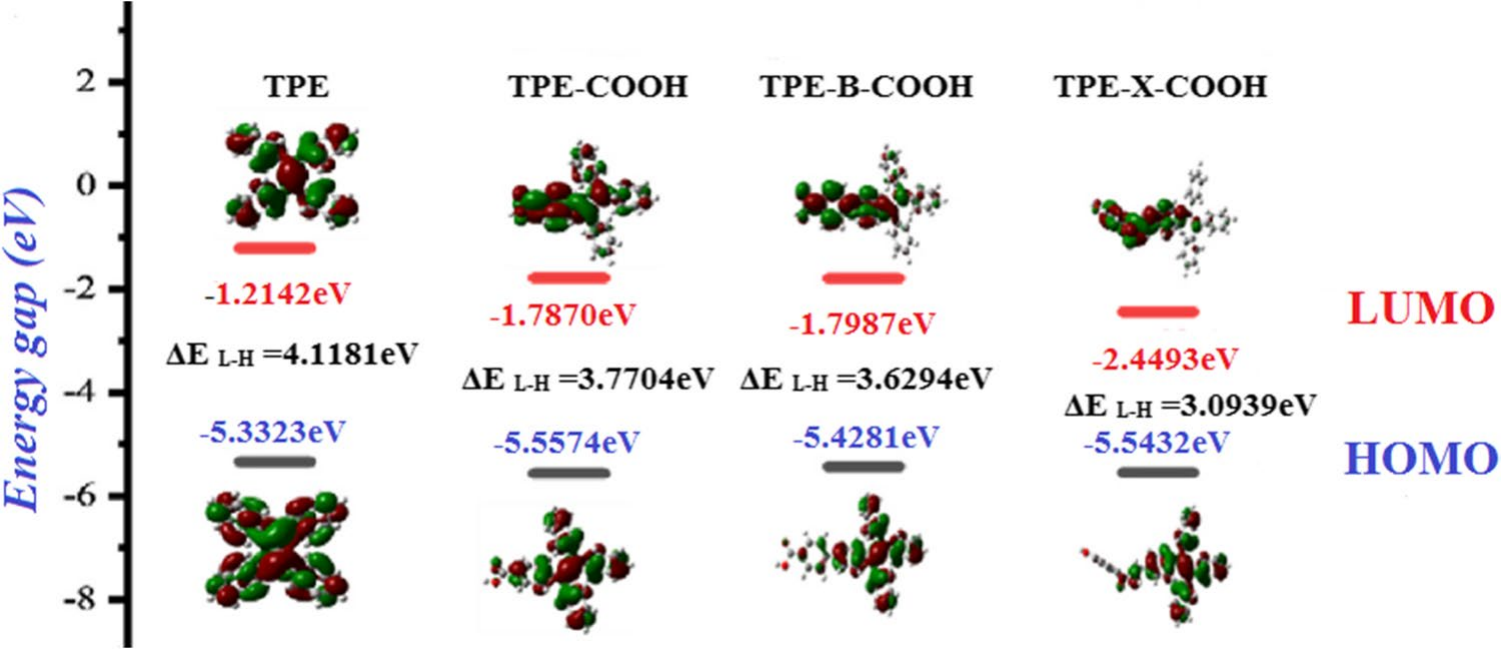
DFT-calculated HOMO and LUMO values of TPE derivative. Reprinted with permission [95]. Copyright 2023, Elsevier

Briefly, Yin et al. designed a portable fluorescence sensor using high-transparency resin and 3D printing technology for sensitive detection of multiple pathogenic bacteria. Using fluorescent probes (tetraphenylethylene (TPE) derivatives) caused bacteria to emit fluorescence with different colors. By injecting fluorescent bacteria into a microfluidic device, the number of fluorescent bacteria was counted. Photosensitive resin was used as the synthetic material for 3D printing. Different fluorescence of three TPE derivatives were proven using DFT computations. Figure 4 presents the results of these computations on TPE and TPE derivatives including TPE-COOH, TPE-B-COOH, and TPE-X-COOH [95].

Litti et al. presented a 3D printed microfluidic chip combined with Janus magnetic/plasmonic nanostars Fe3O4/Au (JMNSs) as surface-enhanced Raman scattering (SERS) colloidal substrates. In this study, several small-molecule analytes were selected, such as a dye with antifungal and antibacterial activity, the standard pH-sensitive molecule p-mercaptobenzoic acid (MBA), a clinically relevant anticancer drug, and herbicide flumioxazin. DFT computations were performed to achieve the Raman spectrum of flumioxazin [96]. Santangelo et al. fabricated a disposable 3D printed lab-on-chip based on epitaxial graphene for heavy metals detection. The 3D printed microfluidic chip interface led to the interaction between the sample solution and the graphene sensing surface. DFT computations were employed for the evaluation of conductivity changes and the sensing mechanism [97]. In spite of the significance of computation and in silico studies and their advantages in decreasing of manufacturing costs, and in the validation of experimental results through accurate computations, however, there are too limited studies about the application of computation in the study and design of 3D printed microfluidic biosensors. In the following, some DFT computations and MD simulations that were carried out to study of 3D printing material are mentioned as a suggestion for more use of these methods in the design of 3D printed microfluidic biosensors. Sun et al. prepared PDMS elastomer with good mechanical strength and self-healing function. They found that their powder-based 3D printing of PDMS elastomer as one the most investigated materials contains hindered pyrazole urea dynamic bonds. Using DFT computations and experiments of small molecule models, they studied the dynamic chemical mechanism of these hindered pyrazole urea bonds [98]. Using a heating-accelerated in situ gelation mechanism and direct ink writing (DIW) technique, Sun et al. reported a strategy to achieve rapid 3D printing of thermosets (silicone Sylgard 184). Heating-accelerated gelation of the thermosetting ink is the underlying principle of their technique. Using DFT computations, they showed that heating reduces the energy barrier for crosslinking and led to the rapid DIW of various thermosetting materials with heterogeneous structures, complex geometries, and multifunctionality [99].

Otieno et al. used both experimental studies and ab initio structural computations to characterize the structural, optical, mechanical, and electronic properties of PLA and ABS. The obtained PLA and ABS via lab-scale filament extrusion showed tensile strengths of 39.07 and 16.12 MPa. In addition, using DFT computations, they achieved the value of 1.899 and 2.539 eV for the band structure of PLA and ABS, and this result indicates the poor conductivity of both materials [100]. Due to properties such as high loading capability and excellent biocompatibility, silica-based mesoporous systems attract attention in drug delivery applications. Pore-size limitation is however one of the challenges in using of these materials to host large molecules such as proteins and enzymes. Considering bone remodeling, Banche-Niclot et al. developed large-pore mesoporous silicas (LPMSs) and used horseradish peroxidase (HRP) as a model enzyme to evaluate the ability of the LPMSs in the adsorption and the release of large biomolecules. By coating of LPMSs with poly (ethylene glycol) (PEG), they indicated the release of biomolecules in response to a pH decrease. Using DFT computations, they determined the pore size distribution of LMPSs and introduced the PEG-coated large mesoporous silica for protein delivery and its application in collagen-based formulation for 3D printing [101]. Tran et al. introduced modified graphene oxide as a gas barrier using polymer epoxy on 3D printing ABS substrates. Using experimental methods such as Raman, atomic force microscopy (AFM), and X-ray photoelectron spectroscopy (XPS), as well as DFT and MD simulations, their graphene epoxy-coated 3D printing substrates exhibited excellent barrier properties for O_2_. They estimated energy barriers of O_2_ in graphene nanopores using DFT computations and studied O_2_ penetration in nanopores using MD simulations [102]. Finding new materials for bone tissue repair is one of the important tasks of modern medicine. For the development of materials, 3D printing offers great opportunities. Stepanova et al. reported the characterization and biological development of 3D scaffolds based on poly (ε-caprolactone) (PCL) loaded with ciprofloxacin or dexamethasone. Preparation of drug-loaded PCL scaffolds by direct 3D printing from a polymer/drug blend was the novelty of their work. They applied DFT computations to calculate the pore characteristics of the 3D printed matrices. The results of computations validated their experimental studies about the effects of drugs on porous characterizes and specific surface area of 3D printed scaffolds [103].

## Challenges and future perspectives

As previously expounded, the utilization of 3D printed microfluidic systems constitutes a paradigm replete with advantages for the meticulous analysis of sweat constituents. Nonetheless, akin to any technological modality, these methodologies are not devoid of inherent challenges. Surmounting these impediments through inventive stratagems possesses the latent capacity to augment the holistic efficacy of the system across multifarious dimensions. A conspicuous challenge resides in the circumscribed array of materials amenable to 3D printing endeavors, with a pronounced emphasis on applications within the purview of biological studies. The imperative necessitates a discerning focus on the exploration and refinement of materials characterized by elevated biocompatibility, particularly in the sphere of implantable devices [104]. Furthermore, the salience of compatibility between these materials and assorted apparatus, such as printed circuit boards (PCBs), cannot be overstated [105]. The integration of hybrid ceramic polymers emerges as a sanguine avenue for augmenting material attributes, especially in contexts mandating optical transparency [106]. Similarly, advancements in mechanical and chemical resistance can be achieved by optimizing curing agent ratios for polydimethylsiloxane (PDMS) molds [107]. The pursuit of new materials and refining existing ones is pivotal for overcoming these challenges and expanding the capabilities of 3D printed microfluidic systems in diverse applications.

The judicious execution of surface modification stands out as a preeminent concern for optimizing the efficacy and dependability of a microfluidic device [108]. Concurrently, the selection of a 3D printing material necessitates meticulous consideration of its compatibility with these surface modifications. A pivotal facet pertaining to both material composition and spatial configuration within the microfluidic framework is the efficacy in fluid collection from perspired exudates. Given the intrinsic variability in the rate of perspiration among individuals and across diverse anatomical regions in the body, the capacity to adeptly harvest a substantial volume of sweat at a designated site augments the sensor’s efficiency by amplifying the quantity of targeted analytes. A pervasive challenge being inherent in 3D printing resides in the dearth of standardized protocols and validated correlations between disparate printing devices and materials. Mitigating this challenge requires a strategic approach, wherein validation endeavors are propelled by ML algorithms that continually scrutinize and adapt to variations in printing techniques, printer parameters, materials employed, and subsequent analysis outcomes post-applications. While advancements in materials science and biosensor technologies remain imperative, complementary impetus can be garnered through robust support for computational studies, thereby fostering a symbiotic relationship between empirical innovation and computational acumen.

## Conclusions

The integration of 3D printing with microfluidics points out a significant advancement in various sensor systems, particularly in the context of sweat analysis. These integrated systems present notable advantages, including expedited manufacturing, cost-effectiveness, and the capacity for continuous monitoring of biomarkers. As materials science progresses and optimization processes refine, 3D printed microfluidic platforms are poised to expand their applicability further. Sweat analysis, on the other hand, emerges as a preferable alternative to blood sample analysis, particularly for populations such as newborns and elderly patients, for whom can be discomforting. The analysis of sweat as a bodily fluid finds applications in disease diagnosis, as well as routine activities like daily walking and sports practice. These integrated systems hold a pivotal position in biomedical research and, notably, disease detection and analysis methods at the home or the point-of-care settings, offering promising prospects for human health. Crucially, for both patients and caregivers, the desirability of wearable systems that enable continuous and autonomous analysis, coupled with the ability to share these analyses via NFC, Wi-Fi, or Bluetooth with devices such as phones or computers, is paramount. Moreover, due to the economic difficulties experienced worldwide, it is essential that everyone has access to such low-cost systems. In addition to accessibility, the easy interpretability of these systems holds great promise for rapid disease detection.

## Data Availability

Not applicable.
